# Unconventional Gas and Oil Drilling Is Associated with Increased Hospital Utilization Rates

**DOI:** 10.1371/journal.pone.0131093

**Published:** 2015-07-15

**Authors:** Thomas Jemielita, George L. Gerton, Matthew Neidell, Steven Chillrud, Beizhan Yan, Martin Stute, Marilyn Howarth, Pouné Saberi, Nicholas Fausti, Trevor M. Penning, Jason Roy, Kathleen J. Propert, Reynold A. Panettieri

**Affiliations:** 1 Department of Biostatistics, University of Pennsylvania Perelman School of Medicine, Philadelphia, Pennsylvania, United States of America; 2 Center of Excellence in Environmental Toxicology (CEET), Airways Biology Initiative, University of Pennsylvania Perelman School of Medicine, Philadelphia, Pennsylvania, United States of America; 3 Department of Health Policy and Management, Mailman School of Public Health, Columbia University, New York, New York, United States of America; 4 Lamont-Doherty Earth Observatory of Columbia University, Palisades, New York, United States of America; Stony Brook University, Graduate Program in Public Health, UNITED STATES

## Abstract

Over the past ten years, unconventional gas and oil drilling (UGOD) has markedly expanded in the United States. Despite substantial increases in well drilling, the health consequences of UGOD toxicant exposure remain unclear. This study examines an association between wells and healthcare use by zip code from 2007 to 2011 in Pennsylvania. Inpatient discharge databases from the Pennsylvania Healthcare Cost Containment Council were correlated with active wells by zip code in three counties in Pennsylvania. For overall inpatient prevalence rates and 25 specific medical categories, the association of inpatient prevalence rates with number of wells per zip code and, separately, with wells per km^2^ (separated into quantiles and defined as well density) were estimated using fixed-effects Poisson models. To account for multiple comparisons, a Bonferroni correction with associations of p<0.00096 was considered statistically significant. Cardiology inpatient prevalence rates were significantly associated with number of wells per zip code (p<0.00096) and wells per km^2^ (p<0.00096) while neurology inpatient prevalence rates were significantly associated with wells per km^2^ (p<0.00096). Furthermore, evidence also supported an association between well density and inpatient prevalence rates for the medical categories of dermatology, neurology, oncology, and urology. These data suggest that UGOD wells, which dramatically increased in the past decade, were associated with increased inpatient prevalence rates within specific medical categories in Pennsylvania. Further studies are necessary to address healthcare costs of UGOD and determine whether specific toxicants or combinations are associated with organ-specific responses.

## Introduction

The United States now leads the world in producing natural gas from shale formations. Shale gas accounted for 40% of all natural gas produced in 2012 [1–4]. In comparison to the early 2000s, natural gas production in the US has increased with more than a 30% increase in production, due in part to the cost-effective combination of horizontal drilling and hydraulic fracturing [1–4].

Unconventional gas and oil drilling (UGOD), including hydraulic fracturing or “fracking”, refers to all activities that extract natural gas and oil from rock formations. At distances from 1 to 2 miles below the earth’s surface, tight rock formations impede natural gas and oil flow into a drill-hole [3]. Common reservoirs that contain natural gas and oils include: porous sandstones, limestones, dolomite rocks, shale rocks, and coal beds. Hydraulic fracturing and horizontal drilling methods can effectively extract these resources. Typically, after drilling is complete, fissures are formed using a perforating gun; a mixture of water, proppants and hydraulic fracturing chemicals is then pumped into the rock [3,5]. Consequently, the fissures remain open to liberate the gas. These substances as well as contaminants released from the shale are present in the flowback water. Contaminants include naturally occurring radioactive materials [3,4], toxic organics and metals that may enter ground water, contaminating water supplies especially if leakage occurs from casement failure or from holding ponds for waste water [6,7]. Other toxicants and volatile organic compounds, such as benzene, ethylbenzene, toluene and xylene or radionuclides, have been seen in ground waters impacted by UGOD spills [8] or surface waters receiving UGOD-related waste water [9]. The general lack of published baseline (i.e., pre-UGOD) data has limited efforts to associate contamination in drinking water wells to UGOD activities [10]. Additionally, exhaust produced by diesel trucks and off-site diesel engines, as well as emissions from other UGOD activities (e.g., venting, flaring, compressor stations, etc.) may also affect local air quality with potential impact on health [11–13]. Plausibly, increased noise pollution, truck traffic, and psychosocial stress due to community change, which occur due to increased hydro-fracking activity, could impact public health [11].

Despite the growth in hydraulic fracturing, the health consequences of UGOD are unclear [3,4,14,15]. In Pennsylvania (PA), a rise in hydraulic fracturing has raised health concerns, especially since the Marcellus Shale formation underlies two-thirds of Pennsylvania [16]. In northeastern Pennsylvania, most wells were drilled for dry gas rather than gas and oil [17]. We postulate that increases in active or producing wells in Pennsylvania from 2007 to 2011 are associated with increases in inpatient prevalence rates. Three counties, which lie on the Marcellus Shale formation along the northern border of PA, were chosen for this study: Bradford, Susquehanna, and Wayne. Importantly, zip codes in Bradford and Susquehanna Counties significantly increased UGOD over this time period. These counties are some of the greatest producers of natural gas in Pennsylvania, generating 489 million cubic feet of natural gas from 598 wells in 2011 [18]. In contrast, zip codes in Wayne County have no active wells [18]. Specifically, we evaluated the association between inpatient prevalence rates and well density within 25 different medical categories, as well as overall inpatient prevalence rates.

## Materials and Methods

This study is an ecological study with the goal of assessing the association between hydro-fracking activity and health care use. Zip code specific inpatient counts were obtained from the time frame of 2007–2011. Only zip codes from the counties Bradford, Susquehanna, and Wayne were considered. For our analysis, only inpatient records for people who resided in one of these three counties were included. Inpatient records of people who came to a hospital in these counties, but did not reside in one of these counties, were excluded. These counties were of particular interest, since Wayne had no hydro-fracking activity between 2007 and 2011, while Bradford and Susquehanna saw increased hydro-fracking activity. Inpatient counts were then converted into inpatient prevalence rates (details in Statistical Methods). Furthermore, for each zip code, we obtained the number of wells for each year in 2007–2011. In total, there were 67 zip codes considered, with five inpatient prevalence rates/well counts each. Inpatient prevalence rates were the primary outcome of interest with wells as the primary predicator of interest.

### Health Utilization Data

Truven Health Analytics (THA) purchased UB92/UB04 inpatient discharge datasets from the Pennsylvania Health Care Cost Containment Council (PHC4). The PHC4 datasets contain all inpatient hospital discharge records, including those for psychiatric and/or behavioral health, rehabilitation, and drug and alcohol treatment, for patients hospitalized in Pennsylvania. Skilled nursing facility (SNF), swing bed, transitional care unit, 23-hour observation, and hospice records are not included. After receipt of state discharge datasets, THA decoded supplied values, checked the validity of information submitted and standardized the format. The ICD-9 diagnosis codes and MSDRGs included in the data pulls can be found in S1 Table, in the supplemental material section.

Truven Health pulled discharge records for patients residing in any of the Bradford, Susquehanna, and Wayne County zip codes for calendar years 2007, 2008, 2009, 2010, and 2011. Treatment records for those patients hospitalized outside of Pennsylvania were not captured. In addition, THA excluded patient records for those patients with dentistry, HIV, and neurosurgery DRGs.

### Insurance Coverage Estimates (ICE) Overview

ICE reports by THA showed the total number of people covered by seven different types of insurance by zip code, age group, and sex for every market in the United States. The seven different types of insurance are Medicaid, Medicare, dual eligible, private employer sponsored, private exchanges, private direct, and uninsured. Every person in a zip code who is a resident is assigned an insurance category based on his or her primary insurance coverage. Only non-residents of zip codes were excluded from the analysis.

### Demographics Methodology

THA acquires all of its demographic data from The Nielsen Company statistics for every zip code in the United States. Nielsen bases their estimates on products of the United States Census Bureau, including the 2010 Census Summary File 1 (SF1). Details of the methodology and definitions used to create the SF1 data, including field definitions and the 2010 Census questionnaire, are available in the Census 2010 Data Definitions publication [19].

### Mapping of Unconventional Gas Wells in Bradford and Susquehanna Counties in Pennsylvania

**Fig 1:**
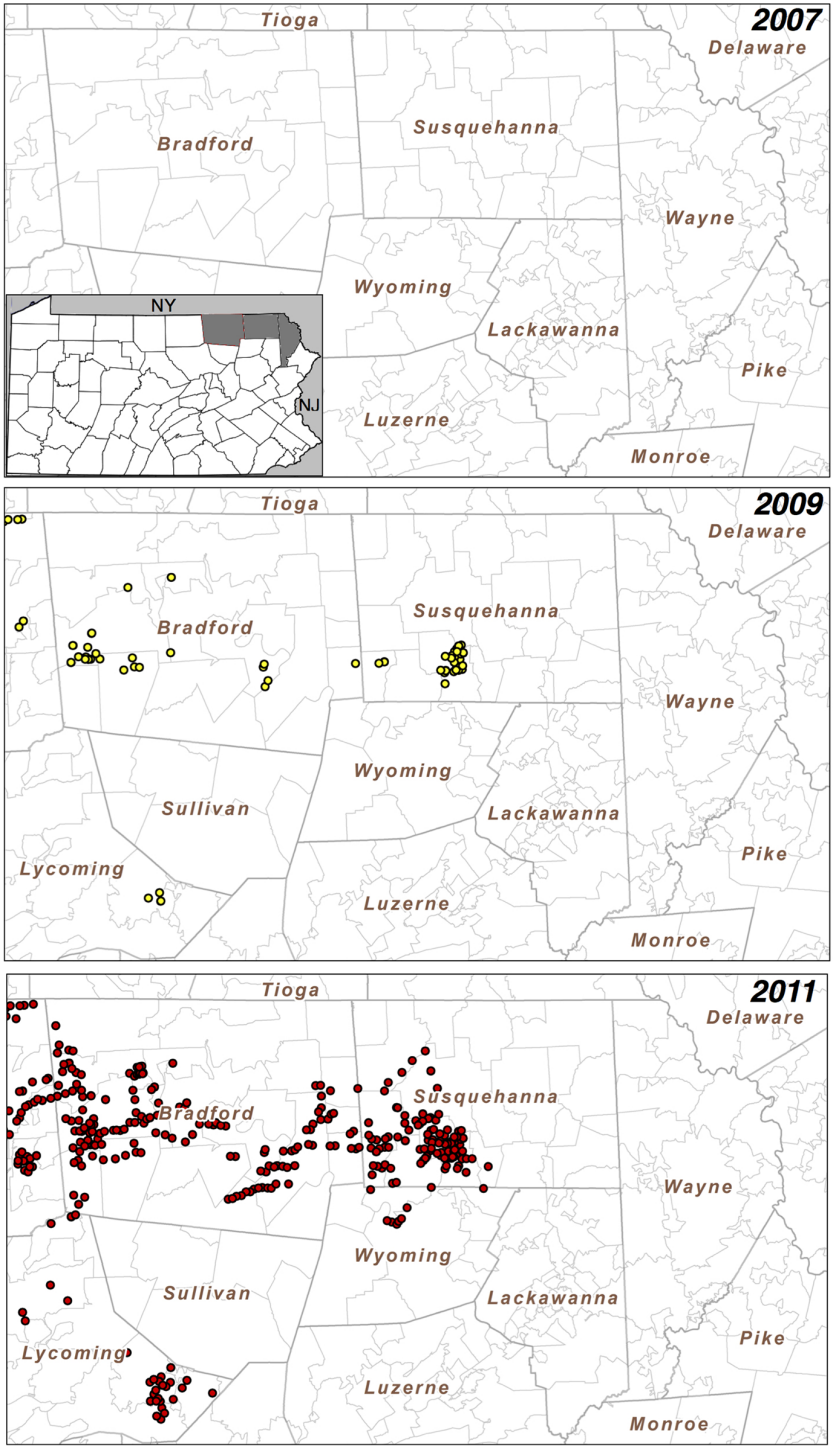
Pennsylvania active wells over time. Pennsylvania active wells in Bradford and Susquehanna Counties increased markedly from 2007 to 2011. Wells are shown as colored dots. From 2007 to 2011, Wayne County effectively had no active wells. Insert in the first panel shows location of Bradford, Susquehanna and Wayne Counties within Pennsylvania.

To create maps of the unconventional gas well locations, the complete data set for 2000–2013 was downloaded as comma separated values (CSV) from the Pennsylvania Department of Environmental Protection Oil and Gas Reporting Website [20] and imported into FileMaker Pro Advanced 13.0.v.3 for further processing. For Fig 1, the data were filtered for unconventional, drilled wells that produced gas in the noted year. We use the state’s categorization, such that: “An unconventional gas well is a well that is drilled into an unconventional formation, which is defined as a geologic shale formation below the base of the Elk Sandstone or its geologic equivalent where natural gas generally cannot be produced except by horizontal or vertical well bores stimulated by hydraulic fracturing.” These data were exported as a DBF file and imported into ESRI ArcGIS v.10.2 to map the locations of the producing wells. In any given year, only wells that produced gas in that year are shown in Fig 1. For example, if a well produced gas in 2007 but did not in 2011, then this well would only appear on the 2007, but not on the 2011 map.

### Statistical Analysis

Statistical analysis was performed using STATA 13 software (StataCorp LP, College Station, Texas). Our data included the number of wells and inpatient counts for all combinations of year, medical category (25 total), and zip code within the three chosen counties in PA. In total, after excluding eight zip codes that had no available population information, 67 zip codes were considered. Only inpatient counts for patients that resided in one of three counties were considered. For each zip code, population and total area per square kilometer (km) data were obtained from the US Census 2010. Importantly, zip code specific population and total area per square km were the same for each year in 2007–2011. Number of wells is defined as the number of wells within a specific zip code for a certain year. All data are generated from active wells. We assume that once a well is active in 2007, this same well remains active for the time frame of 2007–2011. For example, if there are 3 wells in 2007 and 8 wells in 2008 for some zip code, then we assume that there were an additional 5 wells created between 2007 and 2008. This is in contrast to the definition of active wells for the mapping, where a well can move from being active to inactive in any given year in 2007–2011. Given the 5-year observation period, very few active wells became inactive. In addition, the actual date of inactivity could not be accurately defined. Furthermore, it is possible that once a well becomes inactive, it could still impact the surrounding community for some period of time. Thus, for the statistical analysis, once an active well enters at any given year, we assume the well remains active for the remainder of the years. In addition to the count of wells, we also generated wells per square km (wells/km^2^), which is the number of wells divided by the total area per square km (at the zip code level); we defined this variable as well density. We analyzed both exposure variables (count and density) because, a priori, it was unclear whether the number of wells or the density of wells would have a stronger association with health outcomes. Zip code specific inpatient prevalence rates for each medical category (and overall) were calculated by dividing the zip code specific number of inpatient counts per year by the population of the zip code. The inpatient prevalence rates were then converted into prevalence rates per year per 100 people and treated as the primary outcome for modeling. We now refer to prevalence rates per year per 100 people when we discuss inpatient prevalence rates.

Since we examined a relatively brief interval of time (2007–2011), we postulated that in a given zip code, inpatient prevalence rates would be relatively stable. Our goal was to obtain an un-confounded estimate of the association between inpatient prevalence rates and wells. However, it is possible that observable or unobservable zip code characteristics will be correlated with wells and inpatient prevalence rates. Accordingly, we used conditional fixed effects Poisson regression, where the fixed effects are the zip codes. This controls for all possible characteristics of the zip codes, both measured and unmeasured, that did not change during the period of observation. Thus, if zip codes that consistently have high rates of inpatient prevalence rates are more likely to have more wells over time, this will be accounted for in the model. Alternatively, if there are zip code-level changes from 2007–2011 that affect the number of wells and inpatient prevalence rates, this model will not account for this. Essentially, our methodology captures the association between and within zip code changes in wells and inpatient prevalence rates. Furthermore, to account for potential over-dispersion, we use robust standard errors [21]. These robust standard errors are cluster-robust estimates, where the clusters are the individual zip codes in this case. Two sets of analyses are then done to investigate the relationship between inpatient prevalence rates and wells.

The first set of analyses relates inpatient prevalence rates to number of wells. Exploratory analyses suggested that the relationship between the log of the inpatient prevalence rates (Poisson model uses a log link) and number of wells was linear. Thus, for these analyses, prediction variables were the number of wells and year (2007–2011). This assumes a linear relationship between number of wells and inpatient prevalence rates, as well as a linear association between inpatient prevalence rates and year. Note that the primary predictor of interest was the number of wells. This will be referred to as the ***number of wells analysis***.

Furthermore, while exploratory analyses suggested a linear relationship between the log of inpatient prevalence rates and number of wells, we also reasoned that a quadratic relationship between the log of inpatient prevalence rates and number of wells was plausible. Subsequently, we also examined whether there exists a non-linear relationship between number of wells and inpatient prevalence rates. Accordingly, a second model incorporated a quadratic relationship between number of wells and inpatient prevalence rates, for each medical category and overall. Prediction variables within this model were year (2007–2011)/wells, and wells^2^.

The second set of analyses relates inpatient prevalence rates to wells/km^2^ (well density). However, the relationship between inpatient prevalence rates and well density is highly non-linear and heavily influenced by observations that have extremely high wells/km^2^. For example, one zip code located in Bradford had 16.9 wells/km^2^ and 23.4 wells/km^2^ in 2010 and 2011, respectively, while 99% of all wells/km^2^ observations had fewer than 4.28 wells/km^2^. Subsequently, we opted to separate wells/km^2^ into four levels based on quantiles as shown in Table 1. We set Q0wells to be the reference category and all the other levels (Q1wells, Q2wells, Q3wells) to have separate dummy variables. ***This will be referred to as the quantile analysis***.

**Table 1:** Definition of quantiles by wells/km^2^.

	Q0wells	Q1wells	Q2wells	Q3wells
wells/km^2^	0	(0, 0.168]	(0.168, 0.786]	>0.786
Quantile	(0, 65.97]	(65.97, 80]	(80, 90.15]	(90.15, 100]

**Note:** (A, B] indicates that A is excluded from the range, and B is included.

Our analysis investigates the association of increasing wells/km^2^ on inpatient prevalence rates, while allowing for separate associations depending on the magnitude of well/km^2^. We, however, recognize that by using quantiles, we lose information and cannot make inference on explicit changes in well density. Furthermore, while our cut-offs are somewhat arbitrary, the goal is to determine whether increased well density is positively associated with inpatient prevalence rates, which is accomplished by this modeling approach. Overall, the primary predictors for this set of analyses included Q1wells, Q2wells, Q3wells, and year. We test the overall Wald test that the coefficients Q1wells = Q2wells = Q3wells = 0.

For all analyses, risk ratios were obtained by taking the exponential of the regression coefficient estimates. Year is recoded into 2007 = 0, 2008 = 1, 2009 = 2, 2010 = 3, and 2011 = 4. We model each medical category separately as well as the overall inpatient prevalence rates, for a total of 26 models per set of analyses. Furthermore, to adjust for multiple comparisons, we use a Bonferroni correction to adjust for testing 25 different medical categories and overall inpatient prevalence rates in both sets of analyses (52 tests). Using an initial level of significance of 0.05, this means we reject the null hypothesis that wells are not associated with hospitalizations for p<0.00096.

Sensitivity analyses were also performed to determine if removing a specific zip code with much higher inpatient prevalence rates or with much higher well density affected inference. Thus, we removed the specific zip code(s) and recalculated the conditional fixed effects Poisson models, checking to see if the general inference changed.

All of the data obtained for this study were received anonymized and de-identified from Truven Health Analytics. The data were provided as summary information, and there were no unique identifiers. The University of Pennsylvania Committee on the Study of Human Subjects deemed this work non-human subject research.

## Results

### Subject Demographics by County

The three Pennsylvania counties chosen for analysis were Bradford, Susquehanna, and Wayne. These counties were selected given the completeness of health care utilization data from 2007 to 2011. Bradford and Susquehanna Counties also had large increases in active wells over this time period. Wayne County, which effectively had no active wells from 2007 to 2011, served as a unique control population whose demographics were comparable to Bradford and Susquehanna Counties. The total number of residents as per the most recent census in Bradford, Susquehanna, and Wayne Counties was 157,311. As shown in Table 2, the summary of subject demographics for the three Pennsylvania counties obtained from US census data was comparable. Even though the statistical analysis is done at the zip code level, a county level demographic table is an informative summary of the zip codes that are within the counties. Each county is one data point, so no formal statistical comparison is possible. There were no striking differences among the three counties. The subjects were predominantly Caucasian with few people obtaining higher than a high school diploma. Further, the median income was similar among the counties. Table 2 also illustrates the growth in hydro-fracking activity from 2007 to 2011 for Bradford and Susquehanna. By 2011, 95% of the zip codes in Bradford had at least one well, while 70% of the zip codes in Susquehanna had at least one well.

**Table 2:** Characteristics Table for PA Counties.

		Bradford	Susquehanna	Wayne
Population		62,622	43,356	51,548
Overall Hospitalizations 2007–2011		39,821	22,559	30,425
Age (median)		43.4	45.1	45.9
Male %		49.5	50.4	52.8
High School Graduate, percent of person age 25+ %		86.6	88.1	87.4
Bachelor Degree or Higher, percent of person age 25+ %		16.4	16.1	18.4
Median Income (2008–2012) $		44,650	46,815	50,153
Race %	White	97.4	98.0	94.7
	Black	0.6	0.4	3.5
	Asian	0.6	0.3	0.5
	Other	1.4	1.3	1.3
Median Number of Wells	2007	0	0	0
	2008	1	0	0
	2009	13	0	0
	2010	81	1	0
	2011	149	6	0
Number of Zip Codes with >0 Wells (%)	2007	4 (19)	2 (9)	0 (0)
	2008	12 (57)	4 (17)	0 (0)
	2009	16 (76)	8 (35)	0 (0)
	2010	20 (95)	12 (52)	0 (0)
	2011	20 (95)	16 (70)	0 (0)

### Inpatient Prevalence Rates by Medical Category

Table 3 shows the median inpatient prevalence rates and median inpatient counts, along with the interquartile range (IQR), for each medical category as well as overall. The median inpatient prevalence rates and median inpatient counts are to be interpreted at the zip code level. Notably, there are a number of categories with very low (or zero) median inpatient prevalence rates and median inpatient counts. Furthermore, cardiology inpatient prevalence rates/inpatient counts seem to be higher than the other medical categories (excluding overall), with a median cardiology inpatient prevalence rate of 1.99 and a median cardiology inpatient count of 18.

**Table 3:** Median Inpatient Prevalence Rates per 100 people and Median Inpatient Counts, by Medical Category.

Medical Category	Median Inpatient Prevalence Rate (IQR)	Median Inpatient Counts (IQR)
Inpatient total	12.12 (10.05, 14.84)	106 (41, 272)
Cardiology	1.99 (1.42, 2.56)	18 (6, 46)
Dermatology	0.21 (0.09, 0.34)	2 (1, 6)
Endocrine	0.22 (0.01, 0.37)	2 (0.5, 7)
Gastroenterology	1.02 (0.71, 1.43)	10 (3, 27)
General medicine	0.58 (0.32, 0.88)	5 (2, 14)
Generals surgery	0.75 (0.47, 1.01)	6 (3, 19)
Gynecology	0.14 (0, 0.26)	2 (0, 5)
Hematology	0.05 (0, 0.14)	1 (0, 3)
Neonatology	0.12 (0, 0.23)	2 (0, 4)
Nephrology	0.34 (0.18, 0.53)	3 (1, 9)
Neurology	0.58 (0.35, 0.88)	5 (2, 16)
Normal newborns	0.68 (0.41, 0.99)	6 (2, 17)
Ob/delivery	0.84 (0.52, 1.12)	7 (2.5, 21)
Oncology	0.17 (0, 0.29)	2 (0, 6)
Ophthalmology	0 (0, 0)	0 (0, 0)
Orthopedics	1.08 (0.72, 1.42)	10 (4, 26)
Other/ob	0 (0, 0.09)	0 (0, 2)
Otolaryngology	0.08 (0, 0.17)	1 (0, 3)
Psych/drug abuse	0.52 (0.27, 0.85)	5 (2, 16)
Pulmonary	1.18 (0.84, 1.69)	10 (4, 28)
Rheumatology	0 (0, 0.09)	0 (0, 2)
thoracic surgery	0.08 (0, 0.16)	1 (0, 3)
Trauma	0.03 (0, 0.09)	1 (0, 2)
Urology	0.17 (0, 0.27)	2 (0, 5)
Vascular surgery	0.09 (0, 0.19)	1 (0, 3)

**Note:** Median inpatient prevalence rates/median inpatient counts for each medical category and overall are presented, along with the interquartile range (IQR). Median inpatient prevalence rates/median inpatient counts are interpreted at the zip code level.

### Geographic Location of Wells from 2007 to 2011

**Fig 2:**
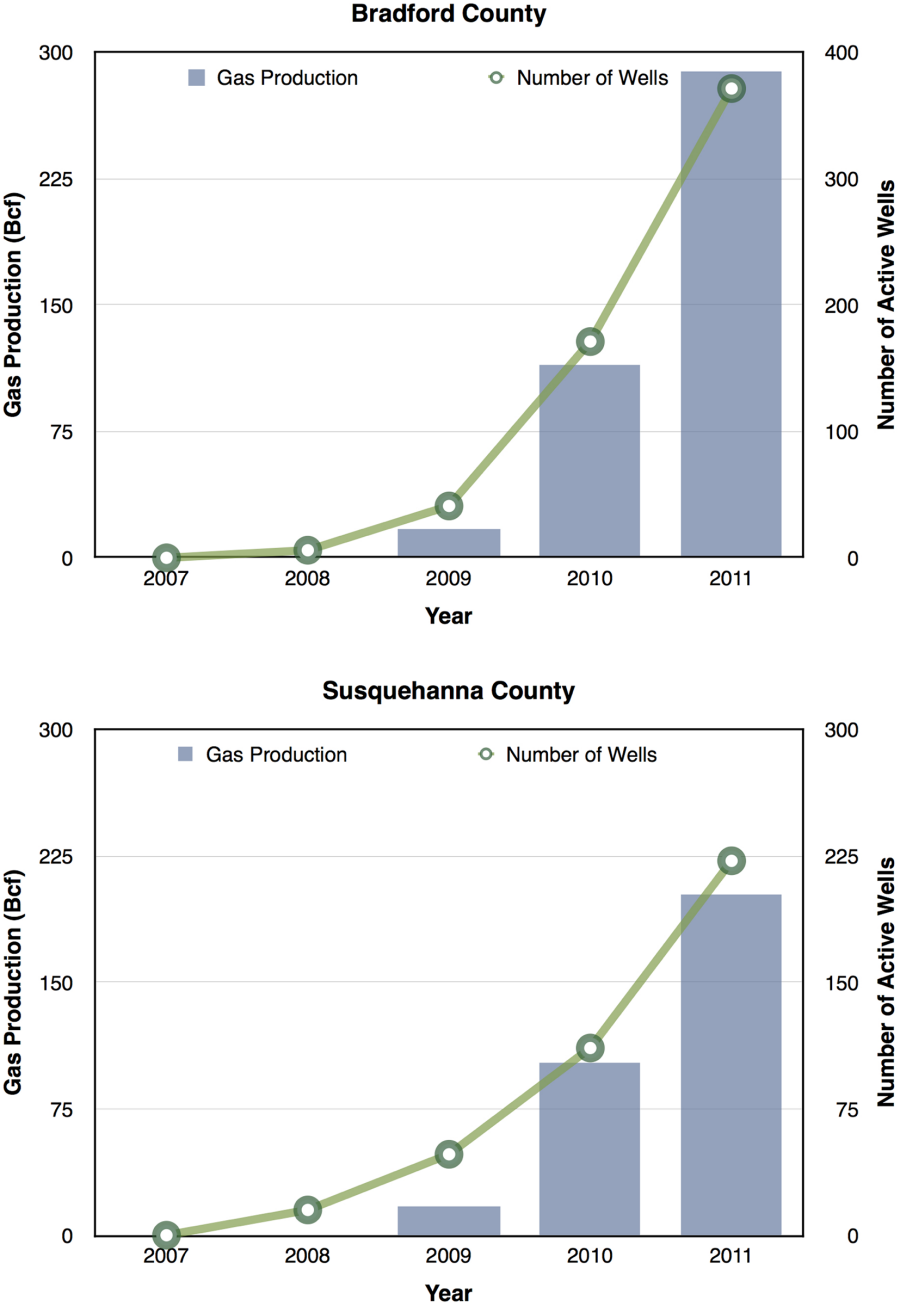
Gas production (histogram) linearly tracked with well number (open circles) from 2007–2011.

Given the demand in accessing the Marcellus Shale for UGOD, we next examined the active wells over time. There was a dramatic increase in the number of active wells from 2007 to 2011 as shown in Fig 1. In Bradford and Susquehanna Counties, there were substantial increases in the total numbers of wells with two zip codes having the greatest number of wells with 400 and 395, respectively. In Wayne County, there were no active wells from 2007 to 2011. The most dramatic increases were in Bradford County where wells were acquired more uniformly than those in Susquehanna County, where active wells were primarily located in the southwest corner as shown in Fig 1. Gas production tracked with increasing active well numbers from 2007–2013 as shown in Fig 2. These data suggest that if UGOD continues at the rates observed between 2007 and 2011, well densities are likely to continue to increase. Within the counties, there were also profound differences in wells by zip code. For example, in 2011, 31 zip codes had no wells, but 17 zip codes had at least 100 wells.

### Increases in Active Wells Are Associated with Increases in Inpatient Prevalence Rates

**Fig 3:**
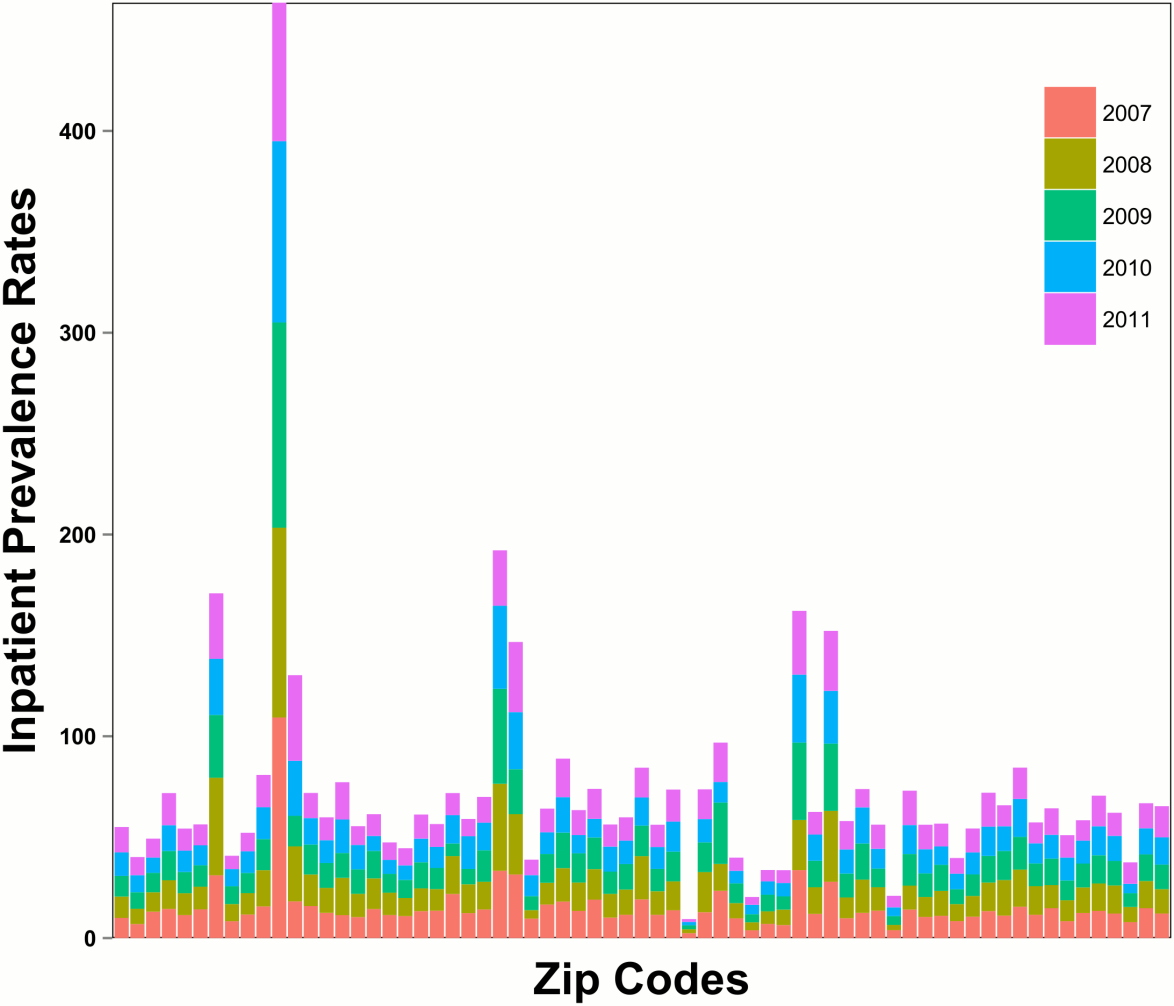
Total inpatient rates by zip code. Total inpatient prevalence rates by zip code. From 2007 to 2011, within a zip code, inpatient prevalence rates are relatively stable.

**Fig 4:**
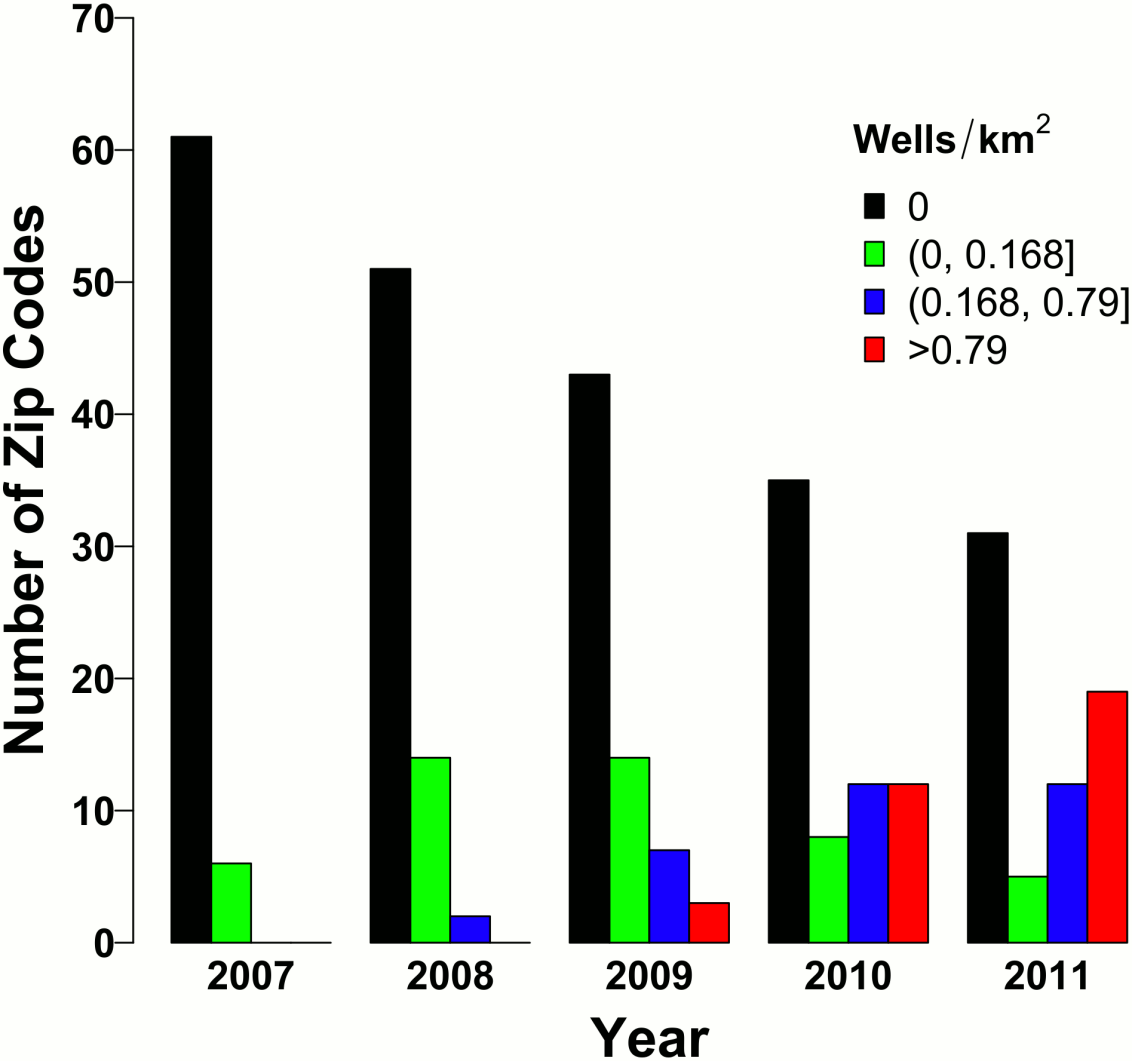
Well density (quantiles) by year. Number of zip codes by well density (quantiles) is presented for each year. In 2007, the majority of zip codes have no wells, but by 2011, the majority of zip codes have at least 1 well.

Given the rapid increase in wells, we reasoned that increases in wells were associated with changes in inpatient prevalence rates. Of the 67 zip codes examined in the three counties, total inpatient counts from 2007 to 2011 were 92,805. There was marked variation in inpatient prevalence rates across zip codes. Specifically, one zip code had a much higher combined inpatient rate as compared with others as shown in Fig 3. Fig 3 also shows that, within each zip code, the contribution by year was comparable, suggesting that within each zip code, the inpatient rates are relatively stable from 2007–2011 Indeed, the average overall inpatient prevalence rates for 2007–2011 are, respectively, 15.18, 15.30, 14.86, 14.00, 14.25. This indicates that on average, zip code overall inpatient prevalence rates were relatively stable or possibly declining from 2007 to 2011, which mirrors national trends [22]. Fig 4 shows how in 2007, 91% (61/67) of zip codes had no wells. However, by 2011, only 46% (31/67) of zip codes had no wells while 54% of zip codes had least 1 well. Notably, many zip codes had a large number of wells by 2011. 28% (19/67) of zip codes had greater than 0.79 wells/km^2^, which equates to 79 wells for every 100 km^2^. Importantly, Fig 4 corresponds to the quantile analysis.

To further understand health consequences by disease category, we modeled the 25 top specific medical categories and total inpatients, investigating the association between number of wells and inpatient prevalence rates and the association between well density and inpatient prevalence rates. Only cardiology inpatient prevalence rates were significantly associated with number of wells, taking into account our Bonferroni correction (p<0.00096) as shown in Table 4. While other medical categories did not strictly meet the Bonferroni correction boundary, a positive association of well number with inpatient prevalence rates within dermatology, neonatology, neurology, oncology, and urology was also evident. Cardiology and neurology inpatient prevalence rates were also significantly associated with well density as shown in Table 5. Furthermore, these results suggest an almost monotonic increase in the impact of well density on cardiology inpatient prevalence rates, considering how the risk ratio increases moving from quantiles (Q1wells to Q2wells to Q3wells). Evidence also suggests that well density was positively associated within the medical categories of dermatology, endocrine, neurology, oncology, urology, as well as overall inpatient prevalence rates (p = < 0.05). Furthermore, for both sets of analyses, the year variable is significantly and negatively associated with inpatient prevalence rates, within the medical categories of gynecology and orthopedics.

**Table 4:** Poisson Fixed Effects Models: Number of Wells per Zip Code per Year.

	Wells RR (p-value)	Year RR (p-value)
Inpatient total	1.0003 (0.076)	0.984 (0.128)
**Cardiology**	**1.0007 (0.0007)**	**0.966 (0.029)**
Dermatology	1.0010 (0.039)	0.977 (0.345)
Endocrine	1.0008 (0.086)	0.963 (0.316)
Gastroenterology	1.0003 (0.338)	0.992 (0.749)
General medicine	1.0002 (0.574)	1.037 (0.022)
Generals surgery	1.0000 (0.849)	1.104 (0.213)
Gynecology	1.0002 (0.708)	0.860 (<0.0001)
Hematology	0.9997 (0.657)	1.023 (0.616)
Neonatology	1.0014 (0.018)	0.959 (0.125)
Nephrology	0.9998 (0.461)	1.025 (0.250)
Neurology	1.0006 (0.037)	1.001 (0.948)
Normal newborns	1.0000 (0.969)	0.963 (0.030)
Ob/delivery	1.0002 (0.411)	0.968 (0.411)
Oncology	1.0015 (0.004)	0.956 (0.081)
Ophthalmology	1.0010 (0.593)	1.084 (0.255)
Orthopedics	0.9993 (0.011)	0.970 (<0.0001)
Other/ob	1.0003 (0.727)	0.899 (0.007)
Otolaryngology	1.0000 (0.982)	0.978 (0.614)
Psych/drug abuse	1.0004 (0.073)	1.035 (0.006)
Pulmonary	1.0000 (0.850)	0.989 (0.482)
Rheumatology	1.0014 (0.043)	0.961 (0.227)
thoracic surgery	1.0011 (0.100)	0.989 (0.708)
Trauma	1.0008 (0.174)	1.021 (0.505)
Urology	1.0010 (0.012)	0.983 (0.464)
Vascular surgery	0.9997 (0.539)	0.948 (0.024)

**Note:** RR = Risk ratio

**Table 5:** Poisson Fixed Effects Models: Quantile Analysis of Wells/km^2^.

	Q1 Wells RR (p-value)	Q2 Wells RR (p-value)	Q3 Wells RR (p-value)	Wald Test of all Q Wells = 0	Year RR (p-value)
Inpatient total	0.979 (0.475)	1.069 (0.044)	1.108 (0.041)	P = 0.0058	0.977 (0.013)
**Cardiology**	**1.021 (0.667)**	**1.142 (0.018)**	**1.27 (0.001)**	**P = 0.0008**	**0.957 (0.004)**
Dermatology	1.051 (0.572)	1.108 (0.429)	1.454 (0.013)	P = 0.0329	0.972 (0.329)
Endocrine	0.975 (0.862)	1.228 (0.045)	1.391 (0.029)	P = 0.0068	0.942 (0.039)
Gastroenterology	0.943 (0.369)	1.12 (0.168)	1.105 (0.364)	P = 0.1101	0.98 (0.406)
General medicine	0.911 (0.234)	0.993 (0.931)	0.985 (0.872)	P = 0.6373	1.037 (0.006)
Generals surgery	0.875 (0.011)	0.921 (0.228)	0.944 (0.424)	P = 0.0669	1.015 (0.157)
Gynecology	0.887 (0.300)	0.938 (0.606)	0.967 (0.849)	P = 0.7549	0.865 (<0.0001)
Hematology	1.202 (0.365)	1.21 (0.320)	1.221 (0.429)	P = 0.7145	0.993 (0.868)
Neonatology	0.994 (0.975)	1.301 (0.152)	1.527 (0.100)	P = 0.0745	0.95 (0.052)
Nephrology	1.115 (0.203)	1.143 (0.227)	1.151 (0.211)	P = 0.5566	1.004 (0.871)
**Neurology**	**0.922 (0.344)**	**1.157 (0.048)**	**1.188 (0.062)**	**P = 0.0003**	**0.99 (0.542)**
Normal newborns	0.949 (0.481)	0.978 (0.764)	0.964 (0.731)	P = 0.8980	0.965 (0.064)
Ob/delivery	0.958 (0.524)	1.028 (0.670)	1.029 (0.749)	P = 0.4219	0.956 (0.002)
Oncology	1.217 (0.144)	1.415 (0.028)	1.815 (0.002)	P = 0.0166	0.938 (0.022)
Ophthalmology	0.717 (0.381)	1.014 (0.976)	1.116 (0.836)	P = 0.5215	1.099 (0.263)
Orthopedics	0.996 (0.940)	0.981 (0.740)	0.875 (0.130)	P = 0.3591	0.963 (<0.0001)
Other/ob	0.966 (0.885)	1.176 (0.451)	1.264 (0.502)	P = 0.7209	0.879 (0.001)
Otolaryngology	1.052 (0.744)	1.194 (0.412)	1.004 (0.988)	P = 0.5564	0.966 (0.527)
Psych/drug abuse	0.944 (0.307)	0.927 (0.293)	1.13 (0.145)	P = 0.0535	1.039 (0.008)
Pulmonary	1.05 (0.267)	1.097 (0.202)	1.067 (0.572)	P = 0.3050	0.981 (0.306)
Rheumatology	1.091 (0.601)	1.432 (0.159)	1.866 (0.034)	P = 0.0774	0.94 (0.067)
Thoracic surgery	0.872 (0.391)	1.151 (0.470)	1.13 (0.654)	P = 0.0903	0.987 (0.751)
Trauma	0.997 (0.987)	1.057 (0.761)	1.265 (0.222)	P = 0.4373	1.02 (0.562)
Urology	0.827 (0.117)	1.105 (0.462)	1.24 (0.215)	P = 0.0334	0.977 (0.339)
Vascular surgery	1.103 (0.488)	1.052 (0.788)	0.966 (0.857)	P = 0.8116	0.946 (0.030)

**Note:** RR = Risk ratio

In both the number of wells analyses and the well density quantile analyses, cardiology inpatient prevalence rates were significantly associated with wells. Under the quantile analyses, neurology inpatient prevalence rates were also significantly associated with well density. Also, both sets of analyses show evidence that dermatology, neurology, oncology, and urology inpatient prevalence rates were positively associated with wells. While only the number of wells analyses showed evidence of a positive association between wells and neonatology inpatient prevalence rates, our findings are consistent with other reports suggesting that such illnesses are linked with hydro-fracking [12].

A quadratic association between number of wells and inpatient prevalence rates was also explored. A quadratic relationship seemed to fit the data better than a linear relationship between number of wells and inpatient prevalence rates, within the ophthalmology and neurology categories, where the p-value for the quadratic number of wells term was, respectively, 0.04 and 0.004. However, these did not meet the Bonferroni threshold. Furthermore, given Table 3 and the sparsity of ophthalmology inpatient prevalence rates (first three quartiles have no inpatient prevalence rates), it seems unlikely that inference is valid for the ophthalmology models. Given this weak evidence of a quadratic association, results for the quadratic number of wells models are not shown.

In our analysis, one particular zip code had extremely high inpatient prevalence rates compared to other zip codes. Thus, a sensitivity analysis was performed (data not shown). This zip code is located within Wayne County and had no active wells from 2007 to 2011. Removal of this zip code from the analysis had little effect on either the number of wells or the quantile analyses, and there was no change in inference and the estimated risk ratios. Next, a zip code in Bradford had extremely high wells/km^2^ in 2010 and 2011, 16.9 wells/km^2^ and 23.4 wells/km^2^, respectively. Consequently, we explored both sets of analyses without this zip code to determine whether removal of this zip code changed inference. Like the first sensitivity analysis, removal of the Bradford zip code had little effect on inference.

## Discussion

We posit that larger numbers of active hydraulic fracturing wells would increase inpatient prevalence rates over time due in part to increases in potential toxicant exposure and stress responses in residents evoked by increases in the hydraulic fracturing work force and diesel engine use. We recognize that a five-year observation period may limit our ability to discern a direct impact on health in the surrounding community but may offer an opportunity to assess hospital utilization rates over time. We examined over 95,000 inpatient records, and thus our study, to our knowledge, represents the most comprehensive one to date to address the health impact of UGOD.

Our data suggests that some but not all medical categories were associated with increases in number of wells, along with increases in well density. Specifically, cardiology inpatient prevalence rates were significantly associated with number of wells and well density, while neurology inpatient prevalence rates were significantly associated with well density. We are struck by the finding that these differences were observable within a short period of time from 2007–2011. We show that from 2011–2013 (Fig 2) the number of active wells continues to rise exponentially. Although we do not have health care utilization data for 2012–2013, if our findings persisted into 2012–2013, it is possible that the association between cardiology inpatient prevalence rates and wells could only become stronger as a result of the increased number of wells (relative to 2007–2011).

The precise cause for the increase in inpatient prevalence rates within specific medical categories remains unknown. Given that our modeling approach cannot account for within zip code demographic changes over the study period, it is possible that some increases were due to an increased influx of subjects to a zip code. Since the inpatient prevalence rates were determined for subjects *who resided* within a zip code, transient UGOD workers whose address was not local were excluded. Thus, our data potentially may underestimate hospital use that excluded those who were not Pennsylvania residents. Further, our data were partitioned into active wells but it is impossible to associate a specific toxicant exposure to an increase in a specific disease category requiring hospitalization. Intriguingly, our findings partially support those of other studies performed in Colorado. Colburn et al. observed that more than 75% of the chemicals used during natural gas operations may affect skin and respiratory systems, as well as other organs [23]. Another study in Colorado also supports our findings in neonatology. McKenzie et al. estimate that being within 10 miles of a gas well significantly increased the odds of having a congenital heart defect by 1.3 as well as the odds of having neural tube defects by two-fold, compared to not being within 10 miles of a gas well [12]. A recent study by Lanki et al. determined that living close to busy traffic was associated with increased C-reactive protein (CRP) concentrations, which is a known risk factor for cardiovascular diseases [24]. This supports our results for cardiology, given the increased truck traffic that comes with increased hydro-fracking activity.

Despite our findings that hospitalization use and active well number are directly associated within specific medical categories, there are limitations to our study. Our study examined a relatively short time interval. Whether our findings will be validated over longer periods of observation remains unclear. To have any association within a brief time frame may forebode greater negative health effects over time. Furthermore, with our limited time frame and data, the functional relationship for the association between well density and inpatient prevalence rates was heavily dependent on many extreme values, which make up less than 1% of the total observations. This motivated the quantile analysis. However, there are clear disadvantages to this approach. By partitioning a continuous variable, we inherently lose information. Furthermore, while we can make inference on moving among quantile levels, we cannot make inference for specific increases in well density. The quantile levels were also somewhat arbitrary, characterized as no wells/km^2^, a “low” amount of wells/km^2^, a “medium amount of wells/km^2^, and a “high” amount of wells/km^2^. Another possible limitation is that our analyses only considered a zip code “exposed” to wells if there were wells within that specific zip code. A zip code with no wells, however, could neighbor another zip code that has many wells. Accordingly, the association between wells and inpatient prevalence rates may be underestimated. Future work will incorporate a spatial aspect, such that the proximity to exposure (wells) is better addressed. Another limitation is that this study, given that we use hospital discharge data, does not include any information on morbidity or mortality. However, a future study that assesses the association between morbidity/mortality and wells would be interesting to explore.

Despite these limitations, our findings may have a significant impact on the consequences of UGOD on health care delivery and policy. For the number of wells analyses, it is useful to consider specific increases in wells, given that the risk ratio associated with the number of wells predictor is in terms of a one unit increase in number of wells. Specifically, consider an increase of 25 wells, which is the observed mean number of wells from our data. For example, if some zip code had an additional 25 wells, we would expect cardiology inpatient prevalence rates to increase by 2% for that zip code. Considering the quantile analyses, if a zip code went from having zero wells to having greater than 0.79 wells/km^2^ (79 wells for each 100 km^2^), we would expect cardiology inpatient prevalence rates to increase by 27% for that zip code. If a zip code went from having no wells to having between 0.17 to 0.79 wells/km^2^, we then would expect a 14% increase in cardiology inpatient prevalence rates for that zip code. Notably, 18 zip codes had greater than 0.79 wells/km^2^, primarily in 2010 and 2011, indicating that each of these zip codes could have had an excess of 27% in cardiology inpatient prevalence rates for each year they had greater than 0.79 wells/km^2^. Furthermore, while dermatology and neonatology were not strictly significant after using a Bonferroni correction, there is evidence that dermatology and neonatology inpatient prevalence rates were also positively associated with wells. From the number of wells analyses, if a zip code had an additional 25 wells, we would expect dermatology and neonatology inpatient prevalence rates to increase by 3% and 4%, respectively. Similarly, from the quantile analyses, if a zip code went from having no wells to having greater than 0.79 wells/km^2^, we would expect dermatology inpatient prevalence rates to increase by 45% for that zip code.

For most medical categories and overall, given the non-significant year risk ratios from Tables 4 and 5, inpatient prevalence rates remained relatively stable between 2007 and 2011. However, within the medical categories of gynecology and orthopedics, inpatient prevalence rates are expected to decrease each year by around 13–14% and 3–4%, respectively. Despite this surprising result, it is unclear why gynecology and orthopedics inpatient prevalence rates are decreasing each year. It is unlikely that these decreasing rates are related to the increased hydro-fracking activity.

To put into the context the potential burden of hydro-fracking on cardiology hospitalizations, consider the zip codes which exceeded 0.79 wells/km^2^ (Q3wells). In total, from 2007 to 2011, three zip codes had >0.79 wells/km^2^ in 2009, 10 zip codes had >0.79 wells/km^2^ in 2010, and 18 zip codes had >0.79 wells/km^2^ in 2011. Some zip codes had >0.79 wells/km^2^ in multiple years, and in total, there were 18 unique zip codes that achieved >0.79 wells/km^2^ at least once. Of these 31 year/zip code observations, the mean cardiology inpatient prevalence rate was 2.17, the mean number of cardiology inpatient visits was 44.74, and the mean population was 2190. Given the model results from Table 5, if these same observations had no wells, we would have expected the mean cardiology inpatient prevalence rate to be 2.17/1.27 = 1.71. Thus, the expected mean number of cardiology inpatient visits, assuming the mean population, would be 1.71*2190/100 = 37.46. However, this is a slight simplification, since each zip code has a different population. We omit the zip code specific populations to preserve zip code anonymity, but when using zip code specific populations, the expected mean number of cardiology inpatient visits, if these zip codes had no wells, would be 35.23. This means that on average, for any year that a zip code exceeded 0.79 wells/km^2^, we would expect an excess of 44.74–35.23 = 9.51 cardiology inpatient visits, compared to if there were no wells. Note that this excess is for a single zip code for a single year in which the zip code exceeded 0.79 wells/km^2^ (this occurred 31 times). A similar exercise shows that for zip codes in the Q2wells range (36 observations total), we would expect on average an excess of 8.13 cardiology inpatient rates. This again is for a single zip code for a single year in which the zip code had >0.168 wells/km^2^ but ≤0.79 wells/km^2^. However, from the model results in Table 5, zip codes with >1 well are in general expected to have increased cardiology inpatient prevalence rates, relative to having no wells. With an inpatient stay costing on average $30K, this poses a significant economic health burden to the Commonwealth of PA.

In summary, hydraulic fracturing as determined by well number or density had a significant association with cardiology inpatient prevalence rates, while well density had a significant association with neurology inpatient prevalence rates. While the clinical significance of the association remains to be shown, UGOD has just begun in Pennsylvania, and thus observing a significant association over this short time is remarkable. Further studies are warranted to compare toxicant exposure to number of wells and inpatient and outpatient studies. Our study also supports the concept that health care utilization should be factored into the value (costs and benefits) of hydraulic fracturing over time.

## Supporting Information

S1 Table ICD-9 diagnosis codes and MSDRGs used in this study. These data are partitioned into three tabs: ICD-9 diagnosis codes, MSDRGs and MSDRG product lines included.
(XLSX)
