# Correction: Unconventional Gas and Oil Drilling Is Associated with Increased Hospital Utilization Rates

**DOI:** 10.1371/journal.pone.0137371

**Published:** 2015-08-28

**Authors:** Thomas Jemielita, George L. Gerton, Matthew Neidell, Steven Chillrud, Beizhan Yan, Martin Stute, Marilyn Howarth, Pouné Saberi, Nicholas Fausti, Trevor M. Penning, Jason Roy, Kathleen J. Propert, Reynold A. Panettieri

The Data Availability statement is incorrect. The correct statement is: All relevant data are available via Figshare (http://dx.doi.org/10.6084/m9.figshare.1508617).

## References

[1] Jemielita T, Gerton GL, Neidell M, Chillrud S, Yan B, Stute M, et al (2015) Unconventional Gas and Oil Drilling Is Associated with Increased Hospital Utilization Rates. PLoS ONE 10(7): e0131093 doi:10.1371/journal.pone.0131093 26176544 10.1371/journal.pone.0131093 PMC4503720

